# Curcumin Ingestion Inhibits Mastocytosis and Suppresses Intestinal Anaphylaxis in a Murine Model of Food Allergy

**DOI:** 10.1371/journal.pone.0132467

**Published:** 2015-07-06

**Authors:** Shannon R. M. Kinney, Logan Carlson, Jennifer Ser-Dolansky, Chelsea Thompson, Sagar Shah, Amos Gambrah, Wei Xing, Sallie S. Schneider, Clinton B. Mathias

**Affiliations:** 1 Department of Pharmaceutical and Administrative Sciences, College of Pharmacy, Western New England University, Springfield, MA 01119, United States of America; 2 Pioneer Valley Life Sciences Institute, Baystate Medical Center, Springfield, MA 01199, United States of America; 3 University of Massachusetts Medical School, Worcester, MA 01655, United States of America; Cincinnati Children's Hospital Medical Center, University of Cincinnati College of Medicine, UNITED STATES

## Abstract

IgE antibodies and mast cells play critical roles in the establishment of allergic responses to food antigens. Curcumin, the active ingredient of the curry spice turmeric, has anti-inflammatory properties, and thus may have the capacity to regulate Th2 cells and mucosal mast cell function during allergic responses. We assessed whether curcumin ingestion during oral allergen exposure can modulate the development of food allergy using a murine model of ovalbumin (OVA)-induced intestinal anaphylaxis. Herein, we demonstrate that frequent ingestion of curcumin during oral OVA exposure inhibits the development of mastocytosis and intestinal anaphylaxis in OVA-challenged allergic mice. Intragastric *(i*.*g*.*)* exposure to OVA in sensitized BALB/c mice induced a robust IgE-mediated response accompanied by enhanced OVA-IgE levels, intestinal mastocytosis, elevated serum mMCP-1, and acute diarrhea. In contrast, mice exposed to oral curcumin throughout the experimental regimen appeared to be normal and did not exhibit intense allergic diarrhea or a significant enhancement of OVA-IgE and intestinal mast cell expansion and activation. Furthermore, allergic diarrhea, mast cell activation and expansion, and Th2 responses were also suppressed in mice exposed to curcumin during the OVA-challenge phase alone, despite the presence of elevated levels of OVA-IgE, suggesting that curcumin may have a direct suppressive effect on intestinal mast cell activation and reverse food allergy symptoms in allergen-sensitized individuals. This was confirmed by observations that curcumin attenuated the expansion of both adoptively transferred bone marrow-derived mast cells (BMMCs), and inhibited their survival and activation during cell culture. Finally, the suppression of intestinal anaphylaxis by curcumin was directly linked with the inhibition of NF-κB activation in curcumin-treated allergic mice, and curcumin inhibited the phosphorylation of the p65 subunit of NF-κB in BMMCs. In summary, our data demonstrates a protective role for curcumin during allergic responses to food antigens, suggesting that frequent ingestion of this spice may modulate the outcome of disease in susceptible individuals.

## Introduction

Food allergy is an emerging public health problem worldwide [1–4]. Severe anaphylactic reactions to food products underscore the need for research to better understand the mechanisms by which food antigens stimulate the gastrointestinal tract and impair tolerance to ingested food particles. In addition, there is a need to develop therapeutic agents that either prevent sensitization to food antigens or suppress the allergic response after initiation.

IgE and mast cells play a crucial role in the development of allergic responses to food antigens [2, 5–7]. Patients with food allergies produce elevated levels of allergen-specific IgE and exhibit both eosinophilic and mast cell inflammation in the gastrointestinal tract [7, 8]. Animal models also suggest a prominent role for mast cells and IgE, as we, and others have previously shown [9–13]. Additionally, the allergic phenotype is driven by Th2 cells, producing high levels of the cytokines IL-4, IL-5, IL-9, and IL-13 in the intestinal mucosa [14, 15].

In contrast to the increased rates of food allergy in the West, the incidence of the disease in developing countries is much lower [1]. A number of theories have been proposed to account for this dichotomy in allergic sensitization, including differences in lifestyle, exposure to pathogens, and dietary habits [8, 16–18]. Dietary components, particularly, have the capacity to influence the mucosal immune system and modulate the allergic response. Curcumin (diferuloylmethane, C_21_H_20_O_6_) is a natural product of the spice turmeric (*Curcuma longa)*, and has well-known pharmacologic activities, including anti-inflammatory and anti-oxidant properties, and has beneficial roles in diseases such as cancer[19], Alzheimer’s [20], and diabetes [21]. Numerous studies implicate a prominent role for curcumin in regulation of the immune system, including the modulation of T cells and mast cells [21]. Additionally, a number of reports also suggest that curcumin possesses anti-allergic activity [22–25]. In a model of ovalbumin (OVA)-induced asthma in guinea pigs, exposure to curcumin inhibited bronchial constriction, and airway hyperreactivity to histamine [22]. Similarly, oral ingestion of curcumin in a latex allergy model, diminished Th2 responses, eosinophilia, IgE production, and lung pathology [24]. More recently, curcumin was shown to suppress the development of *Aspergillus fumigatus*-induced airway inflammation, allergic rhinitis, and OVA-induced allergic conjunctivitis as well as allergic inflammation, in murine models [26–30]. Lastly, curcumin suppresses both T cell and mast cell activation in culture, as well as IgE-mediated mast cell degranulation and passive cutaneous anaphylaxis in mice [31, 32]. Taken together, these data suggest that curcumin may have beneficial roles in Th2-driven diseases and may prevent the development of allergy by suppressing Th2 and mast cell function, leading us to hypothesize that curcumin may also play a role in inhibiting the development of food allergy *in vivo*.

Herein, we show that ingestion of curcumin inhibits both mast cell expansion and suppresses intestinal anaphylaxis in a murine model of OVA-induced food allergy. Oral allergen challenge in sensitized BALB/c mice induced a robust IgE-mediated response accompanied by enhanced OVA-IgE levels, intestinal mastocytosis, elevated serum murine mast cell protease-1 (mMCP-1) and acute diarrhea. In contrast, mice exposed to oral curcumin throughout the experimental regimen did not exhibit allergic diarrhea or a significant enhancement of OVA-IgE and intestinal mast cell expansion and activation. Furthermore, allergic diarrhea, mast cell activation and expansion, as well as Th2 responses, were also suppressed in mice exposed to curcumin during the challenge phase alone, despite the presence of OVA-IgE, suggesting that curcumin may be pharmacologically therapeutic in previously sensitized allergic individuals. Further analysis using both *in vivo* and *in vitro* experimental systems demonstrated that the protective effects of curcumin were mediated by inhibition of mast cell expansion and activation, and that prolonged curcumin exposure induced the apoptosis of mast cells in cell culture. Lastly, the attenuation of intestinal anaphylaxis in this model was linked with the inhibition of nuclear factor-kappa B (NF-κB) activation, a well-established target of curcumin’s anti-inflammatory activity [29, 33]. Similarly, curcumin also inhibited the phosphorylation of the p65 subunit of NF-κB in bone-marrow derived mast cells (BMMCs), suggesting that the protective effects of curcumin during allergic responses may be mediated by inhibiting NF-κB activation in activated intestinal mast cells.

## Materials and Methods

### Animals

BALB/c mice were purchased from Taconic Farms (Germantown, NY). All mice were 4 to 12 weeks old and all animal research was approved by the Institutional Animal Care and Use Committee of Western New England University and received the approval number 2014-S1. The research was conducted according to IACUC guidelines. Animals sacrificed for research were euthanized using a compressed source of CO_2_ gas.

### OVA sensitization and challenge protocol

**Fig 1:**
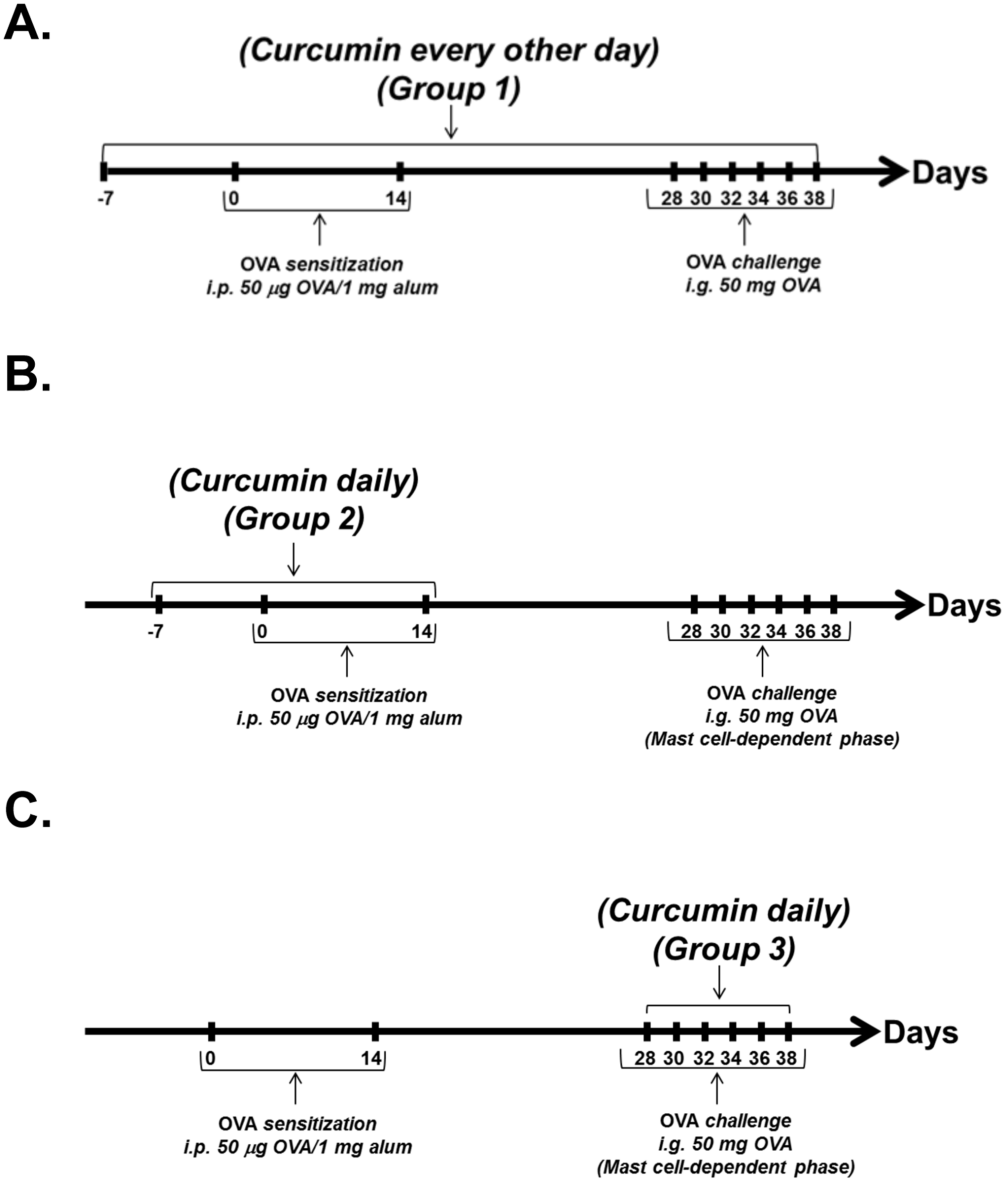
Curcumin treatment protocols. (A) Mice were gavaged with curcumin daily beginning one week prior to sensitization and every other day after the 1^st^ *i*.*p*. exposure. (B) Mice were gavaged with curcumin daily only during the OVA-sensitization phase from days 1–14. (C) Mice were gavaged daily with curcumin during the OVA-challenge phase alone. On days when mice received OVA treatment, curcumin was administered a few minutes later, after all experimental mice received OVA.

To induce food allergy, BALB/c mice were intraperitoneally (*i*.*p*.*)* immunized with 50 μg chicken egg ovalbumin (OVA) in 1 mg alum on days 0 and 14 as previously described [10, 34] and depicted in Fig 1. Two weeks later, mice were challenged intragastrically *(i*.*g*.*)* with 50 mg OVA in 250 μl phosphate buffered saline (PBS) once a day on 6 alternating days. One hour after the 6^th^ challenge, mice were sacrificed and the development of intestinal anaphylaxis was ascertained using previously established procedures [10, 34].

### Curcumin treatment

To determine whether curcumin inhibits the development of food allergy, OVA-primed and challenged mice were gavaged with 300 μg curcumin (Sigma) in 1% carboxy methyl cellulose (CMC) at various times as shown in Fig 1A–1C. Similarly, saline-sensitized, OVA-challenged control mice were also treated with curcumin in 1% CMC. Exposure to oral CMC alone in OVA-primed and challenged BALB/c mice has no effect on the development of intestinal anaphylaxis and mast cell activation (data not shown).

### Measurement of intestinal anaphylaxis

Intestinal anaphylaxis was assessed in challenged mice by scoring the percentage of animals exhibiting allergic diarrhea as previously described [10]. Briefly, mice were observed for the presence of diarrhea for one hour after the 6^th^ OVA-challenge and were scored as diarrhea-positive or negative.

#### Histological analysis and enumeration of mast cells

Intestinal mast cells were enumerated by microscopic examination of sections of paraffin-embedded jejunal tissue as previously described [13]. Tissue sections were stained with chloroacetate esterase (CAE) and mast cells were counted in complete cross-sections of jejunum as specified in the figure legends.

### Quantitative PCR analysis and ELISAs

Quantitative real-time PCR was performed as previously described [13]. Expression of IL-4, IL-5, IL-13, IL-9, IL-10, IL-17, IL-33, and IFN-γ was calculated relative to GAPDH transcripts. mMCP-1 (EBioscience) and OVA-IgE ELISAs were performed on serum samples as previously described [13, 35].

### BMMC culture

BMMC were generated, as previously described [36]. Briefly, marrow was obtained from the femurs and tibiae of naive BALB/c mice, and cultured with 10 ng/ml IL-3 (Peprotech) and 10 ng/ml stem cell factor (SCF) (Peprotech) for 4–6 wks. Harvested BMMC were > 95% positive for c-Kit and FcεRI as detected by flow cytometry.

### Carboxyfluorescein succinimidyl ester (CFSE) labeling and transfer of mast cells

CFSE labeling and mast cell adoptive transfer experiments were performed as previously described [36]. Briefly, BMMC were treated with 5 μM CFSE in culture medium and incubated at 37°C for 5 min without shaking. Approximately 1.0x10^6^ labeled cells were injected *i*.*p*. into BALB/c mice, and their expansion was followed for 6 days. A group of experimental mice was gavaged daily with 300 μg curcumin during this period. On the 7^th^ day, five experimental mice from each group were sacrificed, and peritoneal cells were examined for the presence of CFSE^+^c-Kit^+^FcεRI^+^ cells using an Accuri C6 flow cytometer, and enumerated.

### *In vitro* studies with curcumin

One million BMMCs were cultured in triplicate with IL-3 and SCF or 5 μg/ml DNP-IgE for 6 days. To determine the effects of curcumin on proliferating BMMCs, 30 μM curcumin was added to the cells in dimethyl sulfoxide (DMSO) at the start of the culture. Control wells received DMSO alone. The numbers of total cells/well were counted daily using trypan blue exclusion. To assess the numbers of apoptotic cells, cells were stained with anti-Annexin V and propidium iodide, and the numbers of c-Kit^+^FcεRI^+^ apoptotic cells were determined using an apoptosis staining kit (EBioscience) by flow cytometry as previously described [36].

### β-Hexosaminidase (β-hex) Assay

BMMCs were cultured in triplicate with IL-3 and SCF in the presence or absence of curcumin for 24 h as described above. To induce activation, 2 μg/ml DNP-IgE was added to the cells. 24h later, they were activated with 200 ng/ml DNP-BSA for 1h. β-hex activity was assessed as previously described [37]. Briefly, cells were washed and supernatants and pellets were collected. Pellets were lysed with 0.5% Triton X-100. Both supernatants and pellets were then treated with 4-nitrophenyl-N-acetyl-β-D-glucosaminide (p-NAG) substrate (Sigma) for 1h. Plates were read at 405nm using a spectrophotometer to determine β-hex activity. Data are depicted as percent of cell content demonstrating β-hex activity.

### Assessment of NF-κB activation

Jejunae from experimental animals was fixed in 10% formalin, processed and embedded. Immunohistochemistry for phospho-relA was performed on tissues as previously described [33]. Tissues were sectioned at 4 μm on a graded slide, deparaffinized in xylene, rehydrated in graded ethanols, and rinsed in Tris-phosphate-buffered saline (TBS). Heat-induced antigen retrieval was performed in a microwave at 98°C in 0.01 M citrate buffer. Primary antibody (p- NF-κB Santa Cruz Biotechnology (#sc-101749) 1:400) was exposed overnight followed by secondary anti-rabbit antibody (DAKO (#K4003)). Immunoreactivity was visualized by incubation with chromogen diaminobenzidine (DAB) for 5 minutes. Tissue sections were counterstained with hematoxylin, dehydrated through graded ethanols and xylene, and cover-slipped.

### Western Blot

BMMCs were cultured with IL-3 and SCF in the presence or absence of curcumin for 24 h as described above. To induce activation, 2 μg/ml DNP-IgE was added to the cells. 24h later, they were activated with 200 ng/ml DNP-BSA for 12h. Whole cell extracts were obtained using RIPA buffer containing 1% Triton X-100 and quantified with Coomassie Plus (Bradford) Protein Assay (Life Technologies). Equal amounts of protein were loaded onto 10% SDS-PAGE gels and transferred to PVDF membrane. Membranes were blocked for one hour in 5% milk and incubated with primary antibodies (p- NF-κB (1:500) and Beta Actin (1:2000)) overnight. Membranes were washed with PBS Tween 20 and incubated with appropriate secondary antibody. Membranes were washed a second time, incubated with chemiluminescent reagent (Cell Signaling Technologies), and imaged with a Biorad Chemidoc. Quantification of western blot data was completed using the Biorad Image Lab software. The Volume analysis tool was utilized with local subtraction and resulting values for phospho-relA were divided by the values quantifying beta-actin.

### Statistical Analysis

Data are expressed as mean±SEM, unless stated otherwise. n = 5 mice/group unless indicated otherwise. Statistical significance comparing different sets of mice (between 2 groups) was determined by the Student’s t-test. In experiments comparing multiple experimental groups or time points, two-way analysis of variance was performed and Bonferroni post-tests were used to compare replicate means. P values of <0.05 were considered significant. Analysis was performed using GraphPad Prism software and/or Microsoft Excel.

## Results

### Curcumin ingestion inhibits the development of intestinal anaphylaxis in OVA-exposed mice

We chose to use a well-established model of intestinal anaphylaxis for our studies on the effects of curcumin during allergic responses to food antigens [10, 34, 38]. This model provides a unique system dependent on mast cells and IgE, and OVA-sensitized and challenged animals develop profuse intestinal diarrhea, accompanied by edema and intestinal permeability.

**Fig 2:**
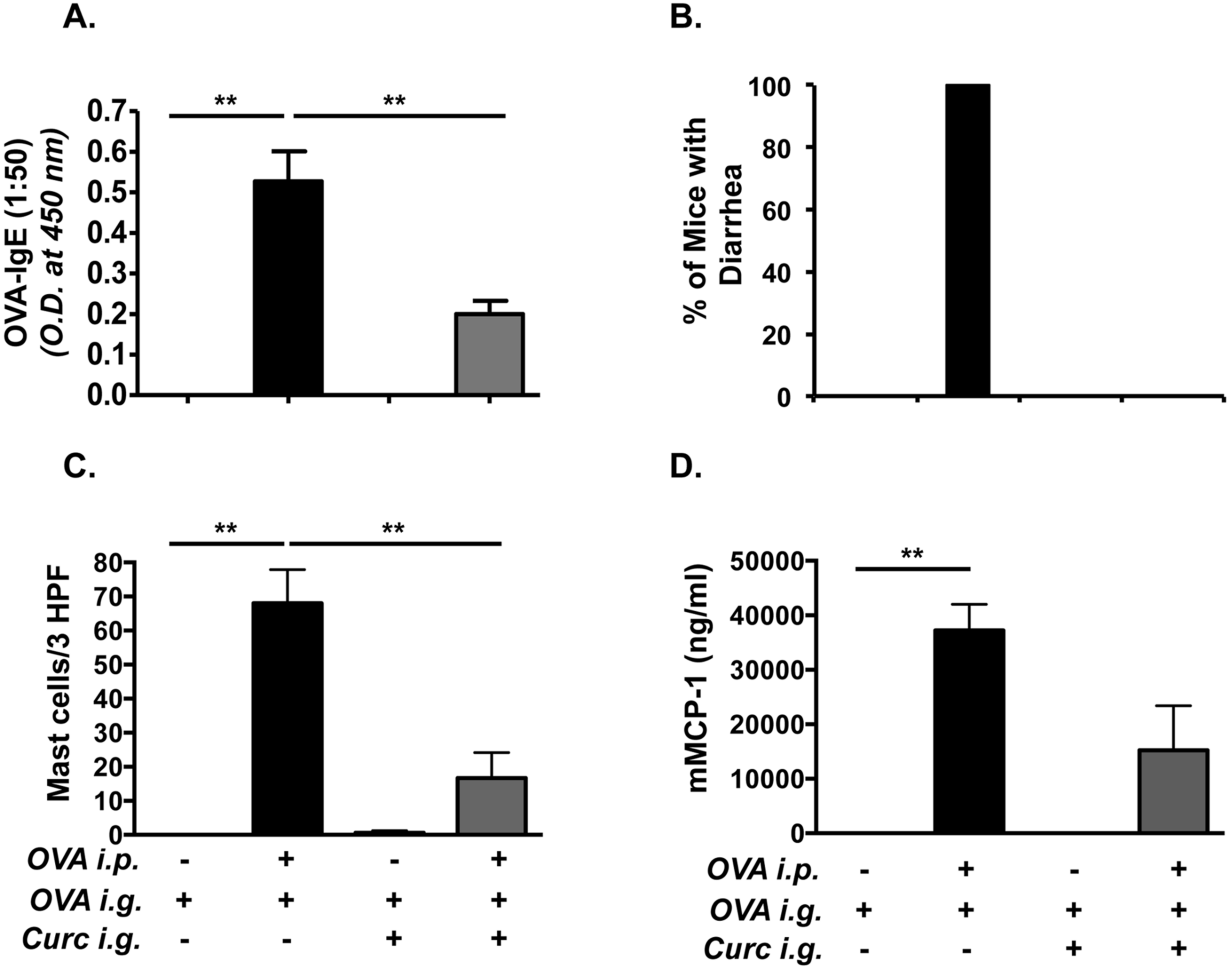
Curcumin ingestion inhibits the development of intestinal anaphylaxis and mastocytosis in BALB/c mice. Mice were sensitized and challenged with OVA and some mice were gavaged with curcumin as depicted in Fig 1A. (A) Serum OVA-IgE levels (1:50 dilution of serum was used for the assay); (B) Percent of mice with diarrhea; (C) numbers of CAE^+^ jejunal mast cells; (D) and serum mMCP-1 levels are shown. Data are representative of 3 independent experiments. ** = p<0.01

Based on studies that curcumin inhibits T cell and mast cell functions, we hypothesized that oral ingestion of curcumin during allergic sensitization and challenge would inhibit the development of intestinal anaphylaxis to food antigens. In order to examine whether curcumin inhibits intestinal anaphylaxis, mice were fed with 300 μg curcumin in 1% CMC daily, beginning one week prior to the first OVA-alum *i*.*p*. challenge, and continued every other day throughout the experimental protocol as depicted in Group 1 (Fig 1A). OVA-challenged BALB/c mice produced elevated levels of OVA-specific IgE compared with saline-treated controls. In contrast, the production of OVA-IgE in OVA-challenged, curcumin-treated mice was significantly diminished (Fig 2A). Similarly, while OVA-sensitized and challenged BALB/c mice exhibited severe profuse diarrhea compared with untreated controls (Fig 2B), none of the OVA-exposed, curcumin-fed BALB/c mice exhibited diarrhea compared with the OVA-challenged, curcumin-untreated group. Likewise, during sacrifice, the production of intestinal edema was also noted in several of the OVA-sensitized and challenged mice, but not in any mice from the curcumin-treated group (data not shown). These data, therefore, suggest that curcumin inhibits the development of IgE production and subsequent intestinal anaphylaxis to oral allergens in this model.

### Curcumin inhibits intestinal mastocytosis in allergic BALB/c mice

The development of allergic diarrhea in this model is dependent on mast cell activation [10]. In light of the above observations, we therefore examined whether curcumin exposure also inhibits mast cell homeostasis in the intestines of allergic mice. Inspection for chloroacetate esterase (CAE) reactivity in the jejunae of OVA-exposed BALB/c mice revealed the presence of numerous mucosal mast cells in the villi compared with saline-exposed controls (Fig 2C). In contrast, the numbers of mucosal mast cells were markedly reduced in OVA-exposed, curcumin-fed animals, suggesting that curcumin inhibits the development of mastocytosis in these animals. In addition, elevated levels of mMCP-1, a marker that has been correlated with an activated mast cell load in tissues, were found in the serum of OVA-challenged BALB/c mice compared with saline controls (Fig 2D). In contrast, the production of this enzyme was decreased (except for one outlier) in OVA-challenged, curcumin-treated mice. These data, therefore, suggest that curcumin inhibits the development of mastocytosis in the intestines of allergic mice.

### The expression of Th2 cytokines is diminished in curcumin-fed allergic mice

**Fig 3:**
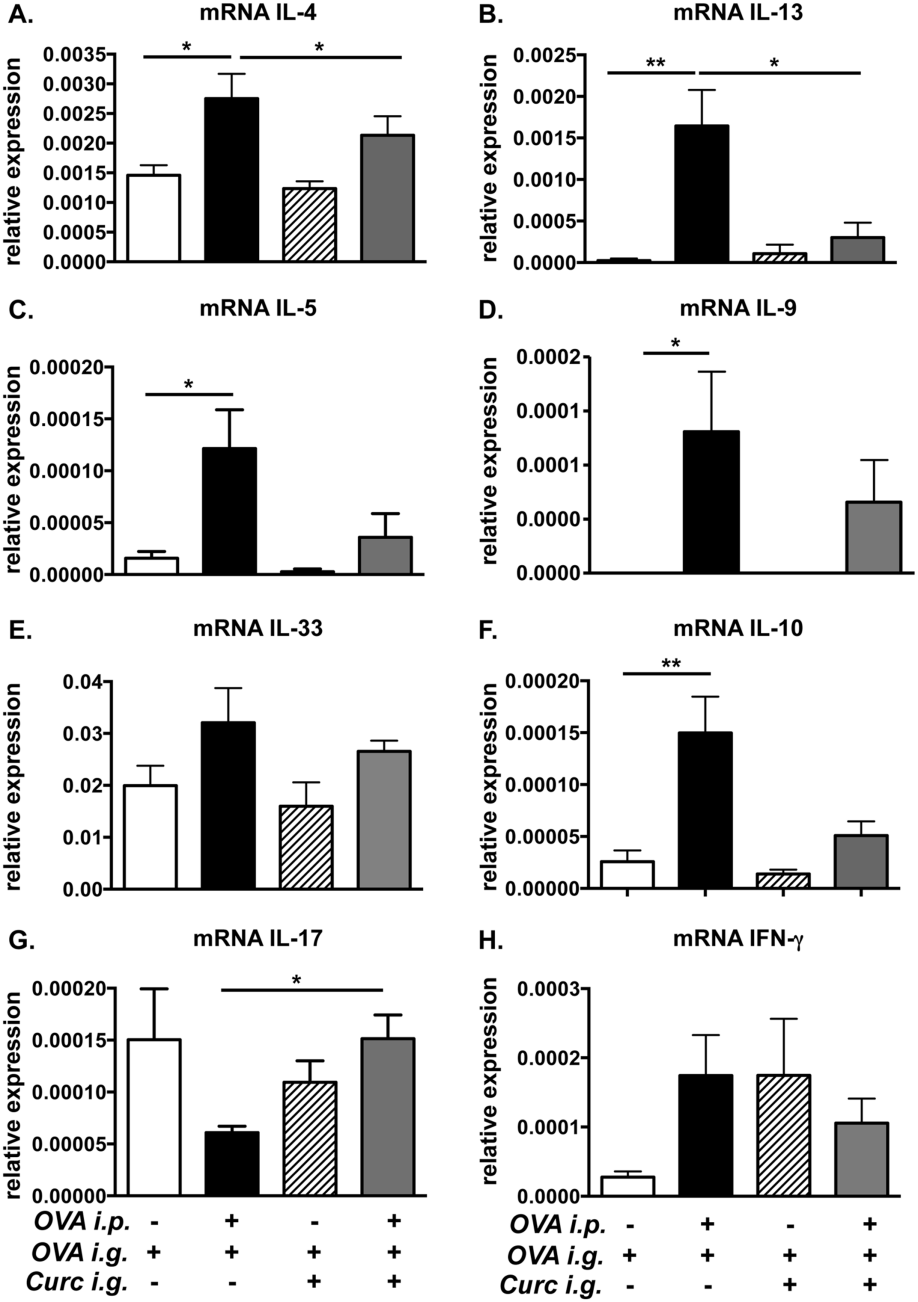
Treatment with curcumin inhibits the expression of intestinal Th2 cytokines in allergic mice. Mice were fed with OVA and curcumin as depicted in Fig 1A. (A-H) Expression of jejunal mRNA for various cytokines is shown. Data are representative of 2 independent experiments. * = p<0.05; ** = p<0.01.

In order to examine whether curcumin suppresses local Th2 responses in the intestines, the jejunae of experimental animals was examined for the expression of the cytokines IL-4, IL-13, IL-5, IL-9, IL-33, IL-10, IL-17, and IFN-γ. As expected, the expression of the classic Th2 cytokines IL-4, IL-5, IL-13, IL-9, and IL-10 was significantly increased in the jejunum of OVA-challenged BALB/c mice compared with saline-treated controls. (Fig 3A–3D and 3F) In contrast, the expression of these cytokines was reduced in the intestines of OVA-challenged, curcumin-treated animals (Fig 3A–3D and 3F). There were no differences in IL-33 or IFN-γ expression in both groups (Fig 3E and 3H). Interestingly however, the increased expression of Th2 cytokines induced by OVA-challenge was also accompanied by a decrease in the expression of the Th17 cytokine, IL-17, in allergic mice (Fig 3G). In contrast, no change in expression of IL-17 was observed in the intestines of OVA-challenged, curcumin-treated mice as compared to saline-sensitized, curcumin-treated control animals (Fig 3G). Taken together, these data suggest that curcumin modulates the T cell response to oral antigen by skewing it away from a Th2-dominated phenotype.

### Oral curcumin ingestion during systemic allergen sensitization attenuates allergic diarrhea but has modest effects on IgE production, mast cell activation, and Th2 responses

The above observations demonstrate a role for curcumin in the inhibition of intestinal anaphylaxis, but it is not clear which aspects of the allergic response are specifically affected. Since mice were exposed to curcumin prior to sensitization as well as throughout the experimental protocol, it is possible that curcumin may have both prevented T cell sensitization to the allergen, and/or inhibited subsequent Th2-dependent responses, including production of OVA-IgE and IgE-dependent mast cell activation. Alternatively, it is possible that the inhibitory effects of curcumin in our model are independent of T cells and are due to suppressive effects on mast cell function during the acute phase of the response.

**Fig 4:**
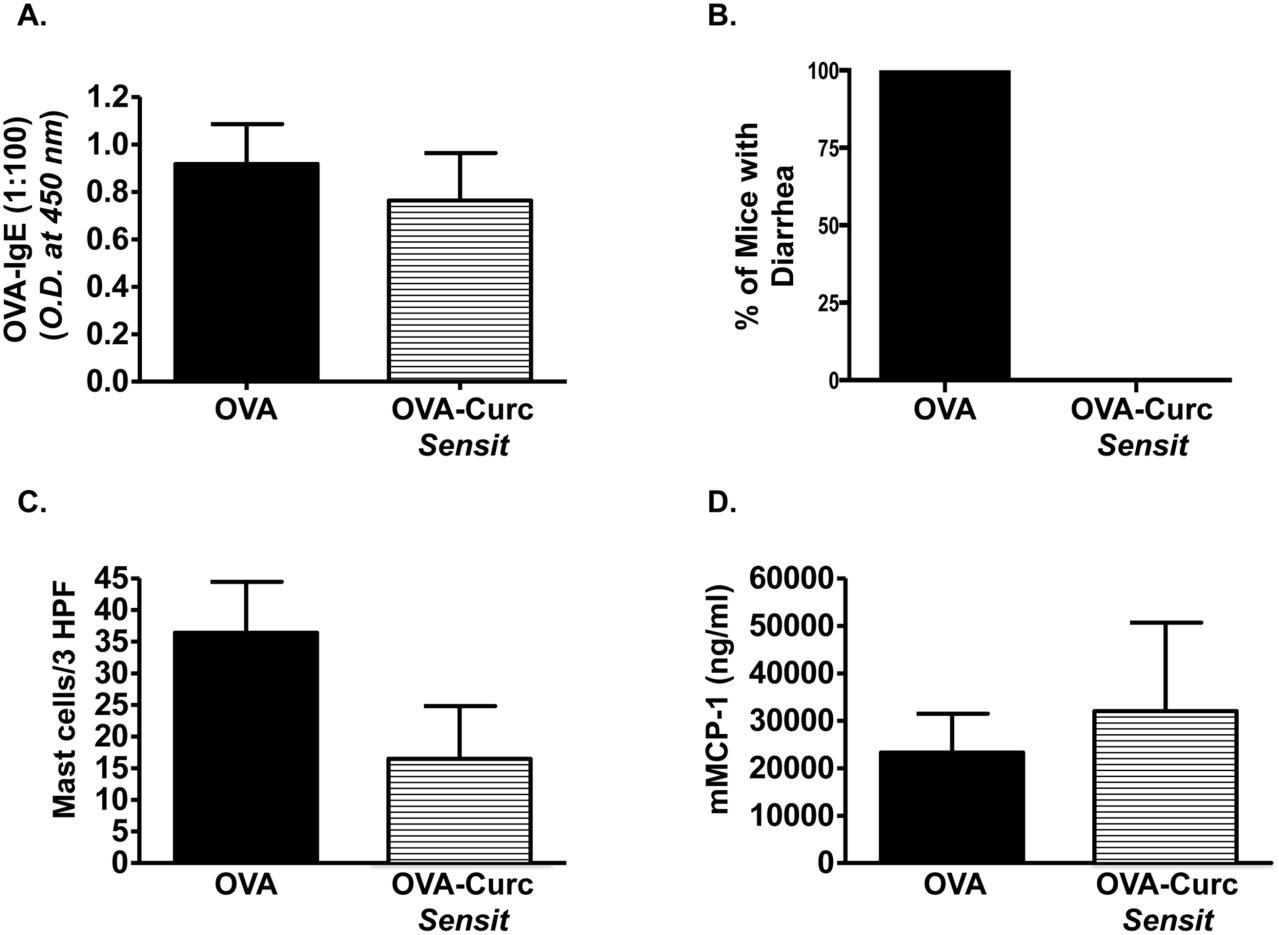
Curcumin exposure during sensitization alone attenuates allergic diarrhea but has modest effects on antibody production and mast cell activation. Mice were fed with OVA and treated with curcumin during sensitization only as depicted in Fig 1B. (A) Levels of serum OVA-IgE (1:100 dilution of serum was used for the assay); (B) Percent of mice with diarrhea; (C) Numbers of CAE^+^ mast cells; (D) and serum mMCP-1 levels are shown.

**Fig 5:**
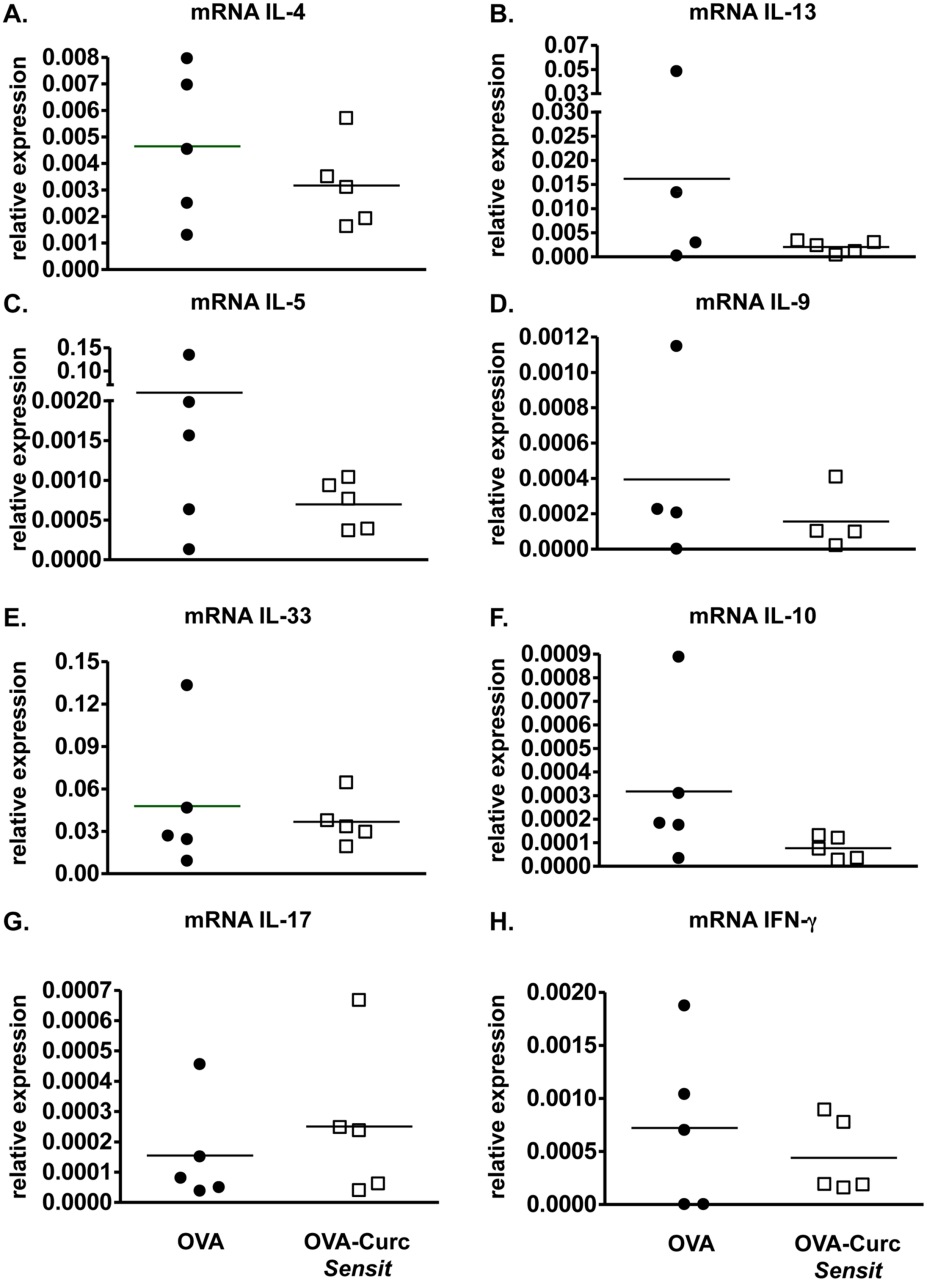
Curcumin exposure during sensitization only results in modest attenuation of intestinal Th2 cytokines. Mice were fed with OVA and curcumin as depicted in Fig 1B. (A-H) Expression of jejunal mRNA for various cytokines is shown.

We therefore assessed whether ingestion of curcumin during OVA sensitization alone was sufficient to inhibit the development of intestinal anaphylaxis. BALB/c mice were sensitized and challenged with OVA, and some mice (Group 2) were gavaged with curcumin only prior to and during OVA *i*.*p*. immunization as depicted in Fig 1B. Ingestion of curcumin during sensitization alone did not attenuate the production of OVA-specific IgE antibodies in allergic mice (Fig 4A). However, while curcumin-untreated, OVA-challenged mice exhibited severe diarrhea in response to OVA challenge, the presence of diarrhea was not observed in the curcumin-treated, OVA-challenged group (Fig 4B). Similarly, while the examination of CAE-stained jejunal sections revealed that intestinal mast cell numbers tended to be lower in curcumin-treated mice, except for 1 mouse (Fig 4C), the levels of mMCP-1 were comparable in both untreated and curcumin-treated mice, suggesting equivalent mast cell activation in both groups (Fig 4D). Lastly, no significant differences in intestinal Th2 cytokine expression were observed between both groups (Fig 5A–5H). These data, therefore, suggest that while curcumin ingestion during OVA-sensitization can attenuate allergic diarrhea and has some protective effects, its effects on antibody production, mast cell activation and Th2 responses is limited, and not sufficient to deliver the full range of pharmacologic benefits as when given during the entire immunization regimen, including both the priming and acute phases of the model.

### Curcumin ingestion during OVA-challenge alone is sufficient to inhibit mast cell-dependent intestinal anaphylaxis despite the presence of OVA-IgE

Mast cells and their mediators drive the acute phase of the response. Furthermore, the effects of mast cells are IgE-dependent in this model [10]. Hence, while it is possible, that any inhibitory effects of curcumin on mast cell activation observed in Groups 1 and 2 may actually be a consequence of inhibition of Th2-dependent OVA-IgE responses, it is likely that curcumin may also have a direct effect on mast cell proliferation and function during the effector phase of the disease. This is suggested by observations that curcumin inhibits mast cell activation and function in cell culture [31, 32], and that it inhibits airway inflammation in a mast cell-dependent model of asthma [29].

**Fig 6:**
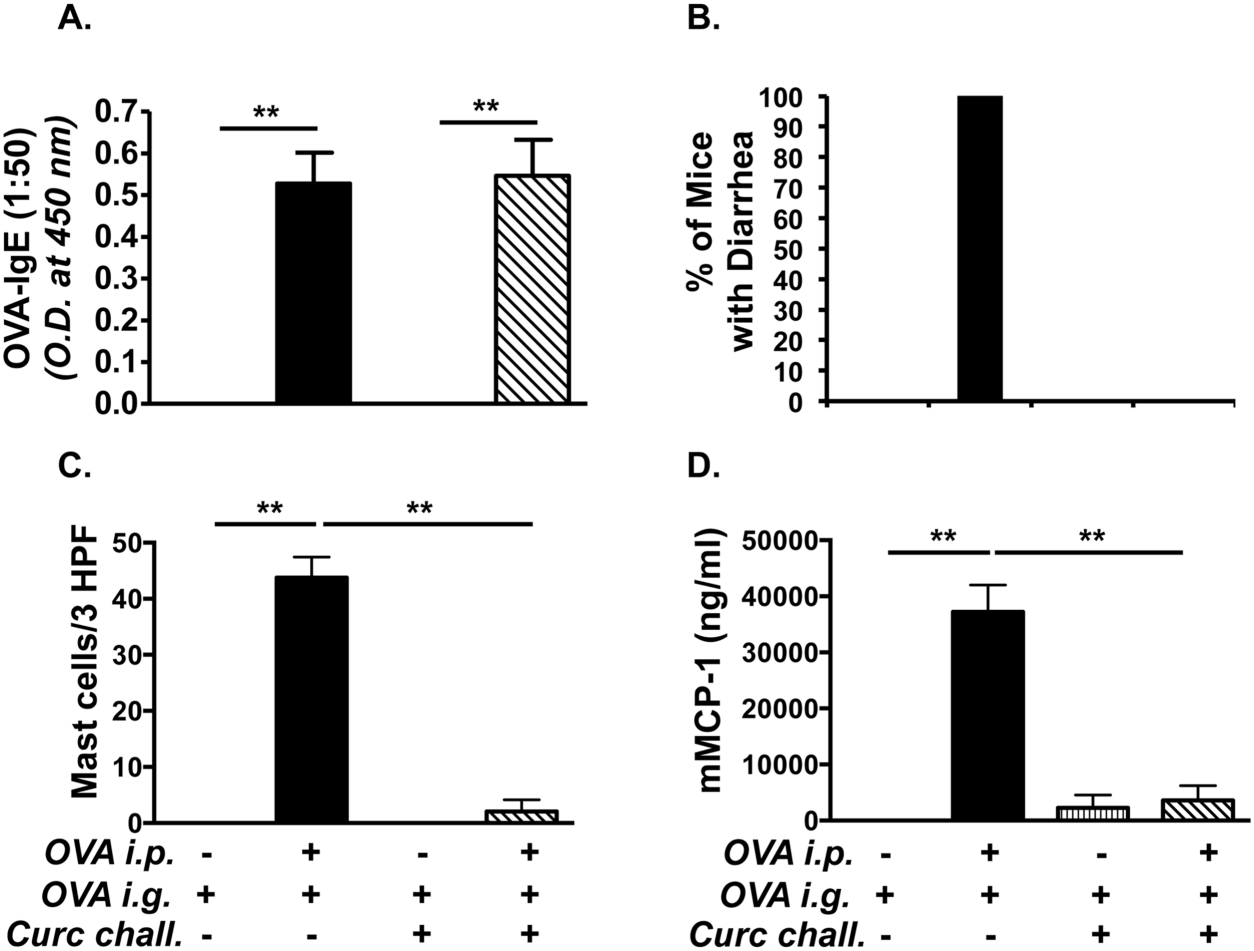
Exposure to curcumin during OVA-challenge alone suppresses allergic diarrhea, and mast cell expansion and activation. Mice were fed with OVA and treated with curcumin during OVA-challenge alone as depicted in Fig 1C. (A) Levels of serum OVA-IgE (1:50 dilution of serum was used for the assay); (B) Percent of mice with diarrhea; (C) CAE^+^ mast cells; (D) and serum mMCP-1 levels are shown. Data are representative of 3 independent experiments. * = p<0.05; ** = p<0.01.

**Fig 7:**
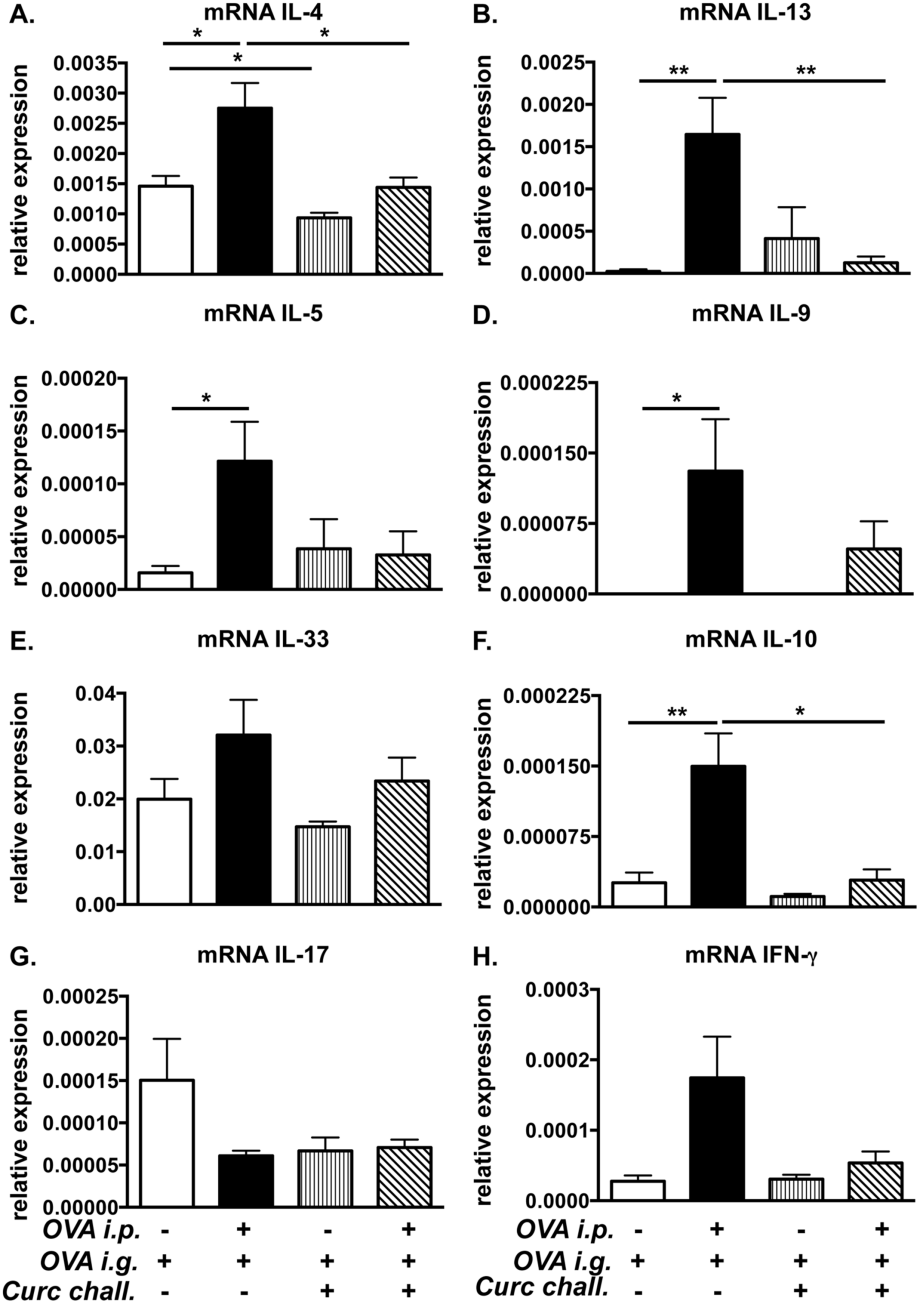
Curcumin treatment during OVA-challenge suppresses intestinal Th2 cytokine production. Mice were fed with OVA and curcumin as depicted in Fig 1C. (A-H) Expression of jejunal mRNA for various cytokines is shown. Data are representative of 2 independent experiments. * = p<0.05; ** = p<0.01.

We therefore examined whether curcumin ingestion during OVA-challenge alone would modulate the outcome of intestinal anaphylaxis in this model. Mice were sensitized and challenged with OVA, and some mice (Group 3) were treated with curcumin during the challenge phase alone as depicted in Fig 1C. OVA-challenge induced the presence of OVA-IgE antibodies in both untreated and curcumin-treated BALB/c mice (Fig 6A), suggesting that curcumin ingestion during OVA-challenge has limited effects on development of Th2 cells and antibody production. Surprisingly, despite the presence of similar OVA-IgE levels, the development of allergic diarrhea was completely inhibited in curcumin-treated mice compared with untreated controls (Fig 6B). Further examination revealed significantly decreased intestinal mast cell numbers (Fig 6C), as well as decreased mast cell activation (Fig 6D), and Th2 cytokine mRNA expression (Fig 7A–7H). These data, therefore, suggest that ingestion of curcumin during the acute mast cell-dependent, OVA-challenge phase can directly modulate mast cell homeostasis and function independent of Th2 sensitization, resulting in the inhibition of mast cell-mediated effects such as allergic diarrhea.

### The expansion of adoptively transferred mast cells is inhibited in curcumin-treated mice

**Fig 8:**
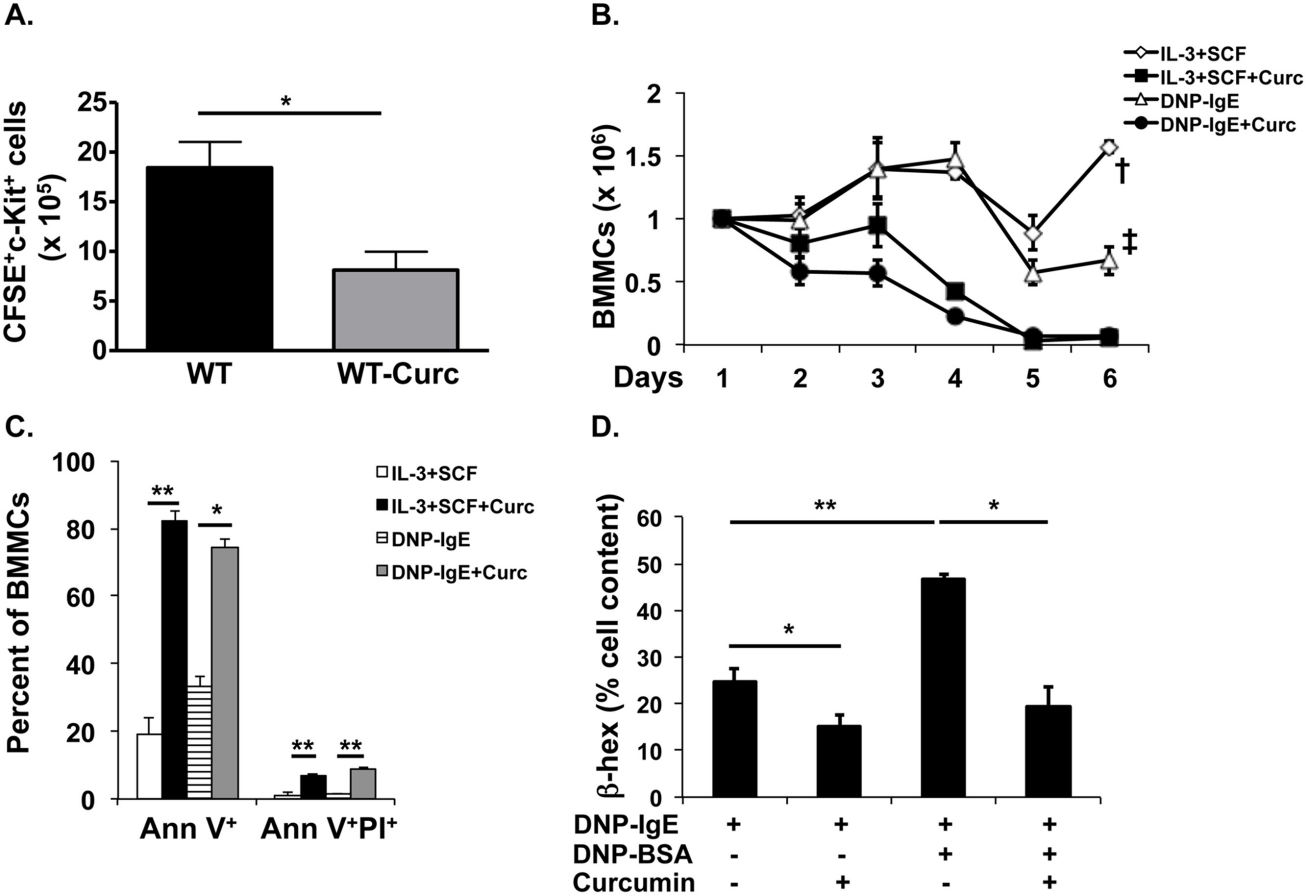
Curcumin inhibits the expansion of mast cells in vivo and inhibits their proliferation, survival and activation *in vitro*. (A) CFSE^+^ BMMCs in the peritoneum of curcumin-gavaged mice. (B-D) BMMCs were cultured in the presence of IL-3 and SCF or DNP-IgE with or without 30 μM curcumin for 6 days. Data are representative of 3 or more independent experiments. (B) Numbers of BMMCs; (C) Percentages of apoptotic BMMCs; (D) and assessment of β-hex activity is shown. * = p<0.05 and ** = p<0.01 by Students t-test. † = p<0.0001 and ‡ = p<0.0005 by ANOVA.

Decreased numbers of mast cells in the intestines of curcumin-treated animals suggests that curcumin blocks the homeostasis of these cells *in vivo* during food allergy. To assess whether curcumin has a direct effect on mast cell expansion, we injected CFSE-labeled BMMCs into the peritoneum of BALB/c mice, and followed their proliferation and survival for six days during curcumin treatments. One week later, the peritoneal lavage was isolated and the numbers of CFSE^+^-mast cells were assessed. As expected, the numbers of CFSE^+^ cells had doubled in the peritoneum of untreated BALB/c mice (Fig 8A). In contrast, a similar expansion of CFSE^+^ cells was not observed in curcumin-treated mice, and in some animals, the numbers of CFSE^+^ cells had decreased from the original number that were injected (Fig 8A). These data suggest that curcumin directly inhibits the expansion of mast cells *in vivo*.

### Curcumin inhibits the proliferation and survival of BMMCs and inhibits their activation

Since curcumin had such a profound effect in inhibiting the development of mastocytosis *in vivo*, we decided to study its effects *in vitro* on the proliferation and survival of BMMCs. BMMCs were cultured with either IL-3 and SCF or DNP-IgE, and their survival was followed as previously described [37, 39]. Strikingly, curcumin completely inhibited the proliferation and survival of BMMCs over a period of 6 days (Fig 8B). Apoptosis studies revealed increased numbers of AnnexinV^+^ cells in cultures treated with curcumin compared to those treated with vehicle alone (Fig 8C), suggesting that curcumin has the potential to induce apoptosis in allergen-sensitized BMMCs *in vivo* and curb their expansion. The β-hex assay was performed to assess the extent of degranulation of activated mast cells. Activated BMMCs exhibited elevated levels of β-hex compared with inactivated controls (Fig 8D). In contrast, treatment with curcumin not only inhibited increases in the levels of β-hex in activated BMMCs, but also suppressed them in IgE-primed, non-activated controls (Fig 8D). The specific release of β-hex was also inhibited by curcumin (data not shown). These data, therefore, confirm the inhibitory effects of curcumin on mast cells observed in the food allergy model, and suggest that its protective effect is mediated by suppression of activated mast cells.

### Treatment of allergic mice with curcumin inhibits the activation of NF-κB in intestinal tissue

**Fig 9:**
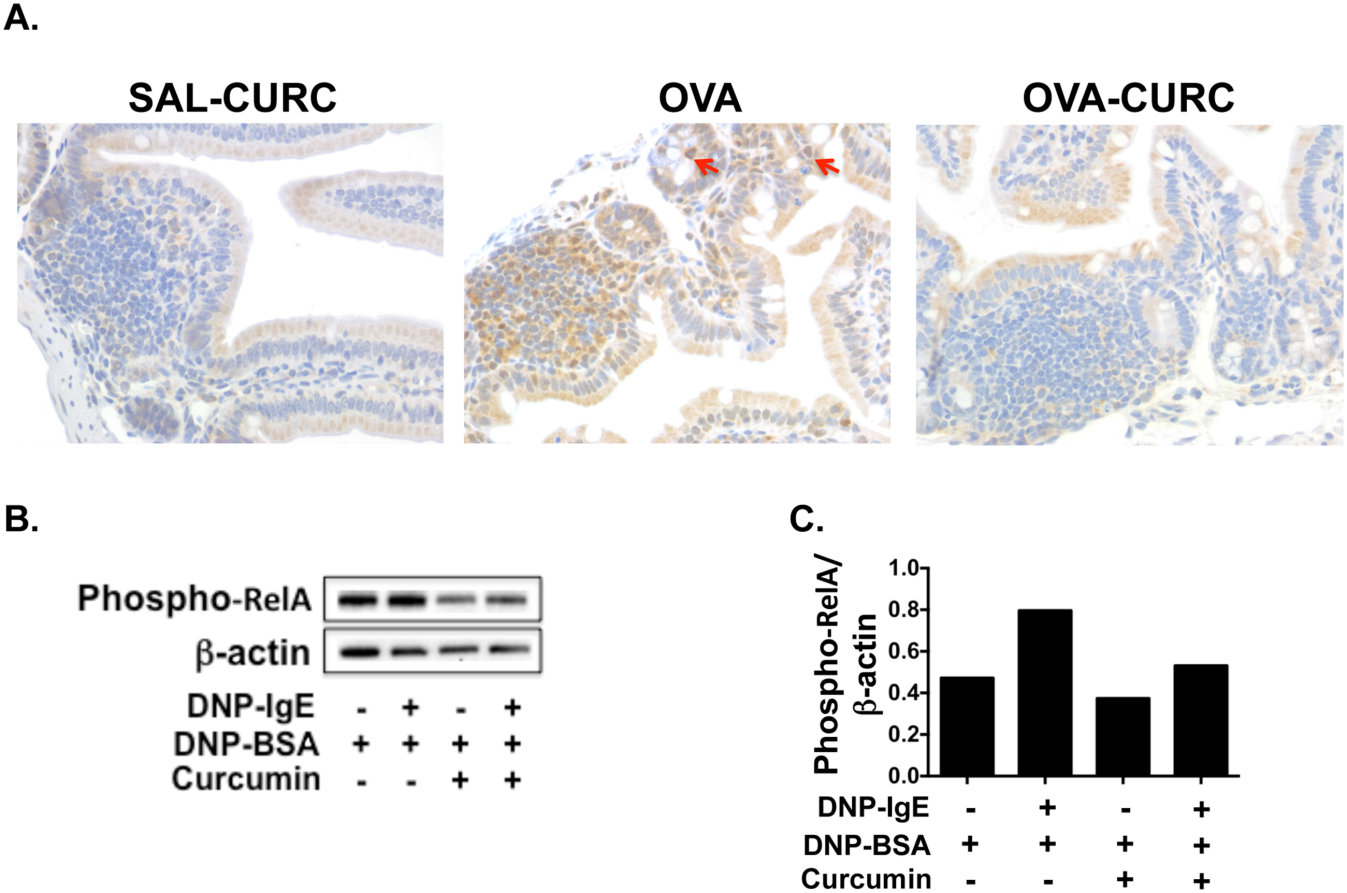
Treatment of allergic mice with curcumin inhibits the activation of NF-κB. (A) Mice were fed with OVA and curcumin and sacrificed as depicted in Fig 1C. Immunohistochemistry on jejunal sections was performed as described in Materials and Methods. Phospho-relA staining (brown) in jejunal tissue is shown. Phospho-relA-positive mast cells as assessed by morphologic analysis are depicted by red arrows. (B) BMMCs were cultured with or without DNP-IgE and 30 μM curcumin in DMSO and activated in the presence of antigen 24 hours later. 12 hours later, protein was extracted from whole cell lysates and Western blot was performed. Data are representative of three experiments. (C) Quantification of the Western Blot data from B is shown.

A number of studies indicate that curcumin is a potent non-specific inhibitor of the transcription factor NF-κB [40–42], which is involved in the activation of both T and mast cells. To determine whether curcumin inhibits NF-κB activation in this model, we assessed the activation of NF-κB in the intestinal tissues of allergic mice (Fig 9A). The p65 (RelA) sub-unit of NF-κB plays a crucial role in the activation of NF-κB and its phosphorylation at Ser^276^ (phospho-relA staining) can be assessed by immunohistochemistry as previously described [33]. While the intestinal tissue of saline-sensitized and OVA-challenged control mice did not exhibit significant phospho-relA staining, the number of phospho-relA^+^ cells (including mast cells as assessed by morphologic analysis) in the intestines of OVA-sensitized and challenged mice was dramatically increased, suggesting that the induction of allergic responses is accompanied by NF-κB activation. In contrast, the intestines of OVA-exposed, curcumin-treated mice appeared to be similar to those of saline-treated controls suggesting that curcumin inhibits the activation of NF-κB. These data, therefore, suggest that the inhibitory effects of curcumin on mast cells in our model may be conferred by blocking the activation of NF-κB.

### Curcumin treatment inhibits the phosphorylation of NF-κB p65 in activated BMMCs

To further assess the effects of curcumin on NF-κB activation in mast cells, we examined the presence of NF-κB p65 (phospho-relA) phosphorylation in BMMCs activated by cross-linking via the IgE receptor and antigens. While activated BMMCs clearly exhibited the phosphorylation of the p65 subunit at Ser^276^, decreased phosphorylation was observed in BMMCs that had been pre-treated with curcumin prior to activation with antigen. Similarly, a decrease in phosphorylation was also observed in curcumin-treated inactivated controls (Fig 9B and 9C). Taken together, these data therefore indicate that the effects of curcumin on mast cell activation and function during allergic responses *in vivo* may be mediated by the inhibition of NF-κB activation in mast cells.

## Discussion

Food allergy is a growing health problem, especially in Western countries, where it has a significant impact on the health and daily activities of allergic individuals. The findings reported here indicate that dietary components such as curcumin, with established anti-inflammatory and anti-allergic activity, can significantly modulate the mucosal immune response and may thus have therapeutic consequences in patients with food allergies.

Numerous scientific studies show beneficial roles for curcumin and it has been investigated as a therapeutic agent in clinical trials for several diseases, including ulcerative colitis [43], inflammatory bowel disease [44], Alzheimer’s [45], and cancer [46]. Curcumin’s effects on the immune system are manifold, and are consistent with its broad pharmacological effects, such as the inhibition of NF-κB, which is involved in the activation of many immune cells. Here, we show that ingestion of curcumin during allergic sensitization and challenge abrogates the development of intestinal anaphylaxis, and inhibits mast cell activation and Th2 responses during food allergy. Furthermore, the protective effects of curcumin were observed in previously sensitized mice and reversed the development of food allergy in allergic mice despite the presence of OVA-IgE antibodies, suggesting a strong potential for therapeutic use of curcumin in allergic patients.

The suppression of polyclonal T cell activation by curcumin has been observed in many disease models [21], and we therefore anticipated that curcumin exposure would inhibit Th2-dependent effects, such as the production of OVA-specific IgE antibodies and the subsequent development of IgE-dependent, mast cell-mediated intestinal anaphylaxis. However, whether the observed effects of suppression of intestinal anaphylaxis were specifically due to inhibitory effects of curcumin on Th2 cells or mast cells was not clear. While curcumin ingestion during OVA sensitization attenuated allergic diarrhea and OVA-IgE, consistent with its effects on suppression of T cells, the effects were modest, and a complete inhibition of the allergic response in terms of mast cell-mediated effects was not observed. This, therefore, suggested that while curcumin has limited effects on allergic sensitization, its protective effects may be conferred during re-exposure to the allergen during the acute phase. Surprisingly, examination of mice treated with curcumin during the acute, OVA-challenge phase, demonstrated a complete attenuation of allergic diarrhea, as well as mast cell activation and Th2 responses. Moreover, inhibition of the allergic response was observed despite the presence of OVA-specific IgE antibodies.

These data, therefore, suggested that the inhibitory effects of curcumin may be a consequence of direct suppression of mast cell activation and function in allergic mice. This was corroborated by observations that the expansion of adoptively transferred BMMCs is limited in the peritoneum of curcumin-treated mice, and that curcumin inhibits the proliferation and survival of BMMCs *in vitro*, and induces their apoptosis. Furthermore, curcumin may also affect mast cell function during allergic responses. Our data demonstrates that curcumin can inhibit mast cell degranulation, as indicated by the release of β-hex, and Lee *et al*. demonstrated that curcumin can inhibit the production of IL-4 and TNF-α by RBL cells [32]. This is consistent with our observations demonstrating the decreased mRNA expression of IL-4 and other Th2 cytokines in the intestines of curcumin-treated animals.

The decreased activation of mast cells in OVA-challenged, curcumin-treated mice during the challenge phase, despite the presence of elevated levels of IgE antibodies, suggests that the suppression of mast cell function by curcumin may occur by affecting targets downstream of the antigen:IgE:FcεRI signaling cascade, and/or as a result of blockade of calcium mobilization as has been previously shown in T cells [47], Our data showing that activation of NF-κB is inhibited in curcumin-treated mice and in BMMCs, is consistent with effects on the signaling pathway, and may directly explain the effects of curcumin on mast cell responses in the model. Additionally, Lee *et al*. demonstrated that the inhibitory effects of curcumin on mast cell activation *in vitro* were due to the inhibition of Syk kinase activity (which likely occurs upstream of NF-κB signaling in mast cells) [32], suggesting that similar effects may also be mediated by curcumin in allergic mice during food allergy. In this context, a recent paper demonstrating that the pharmacologic blockade of Syk reversed the establishment of food allergy [48], confirms that oral treatment aimed at inhibiting Syk activity can ameliorate allergic responses, and suggests that this may be a highly plausible mechanism by which curcumin suppresses mast cell expansion and activation during allergic responses. Lastly, curcumin is known to have a range of pharmacological targets, and its effects on other immune cell types, such as dendritic cells [49] and regulatory T cells [50], may also be important and warrant further investigation in the examination of food-induced allergic responses. In this context, whether curcumin may affect the absorption of OVA by antigen-presenting cells as well as other intestinal cells is also an important question that needs to be further investigated.

In summary, we report novel findings on the anti-allergic effects of a natural dietary compound, curcumin, which may have the potential for therapeutic use in patients with food allergy. Curcumin and its derivatives are already being used in clinical trials, and analogs with better bioavailability could be developed that are more effective in patients with food allergy [19]. Furthermore, our data demonstrates novel inhibitory effects of curcumin on mast cell activation and intestinal anaphylaxis, thus providing further insight into the mechanism by which this natural compound regulates allergic responses. Lastly, our data indicate that dietary substances such as curcumin, which is a daily component of the diet of some countries, have the potential to regulate mucosal immune responses, warranting further investigation into the association between dietary factors and the development of food allergy in nations with higher prevalence of the disease.
